# Secondary oxalate nephropathy and impact of high‐dose vitamin C intake for COVID‐19 prevention on a patient with Roux‐en‐Y gastric bypass: A case report

**DOI:** 10.1002/ccr3.7020

**Published:** 2023-03-07

**Authors:** Fatemeh Kafi, Mojgan Mortazavi, Alireza Pouramini, Shahaboddin Dolatkhah, Behrouz Kaleidari, Diana Taheri

**Affiliations:** ^1^ Department of Pathology, Isfahan Kidney Diseases Research Center Isfahan University of Medical Sciences Isfahan Iran; ^2^ Urology Research Center Tehran University of Medical Sciences Tehran Iran; ^3^ Isfahan Kidney Diseases Research Center Isfahan University of Medical Sciences Isfahan Iran; ^4^ Department of Internal Medicine Iran University of Medical Sciences Tehran Iran; ^5^ Alzahra Hospital Isfahan University of Medical Sciences Isfahan Iran

**Keywords:** COVID‐19, gastric bypass, hyperoxaluria, oxalate nephropathy, prostatic hyperplasia, vitamin C

## Abstract

The current study is important in informing clinicians about the possibility of concurrent oxalate nephropathy caused by Roux‐en‐Y gastric bypass, high oxalate materials, and high‐dose vitamin C intake for COVID‐19 prevention.

## 1 INTRODUCTION

Obesity and COVID‐19 are two of the most threatening pandemics being confronted worldwide. According to the World Health Organization (WHO), 1.9 billion persons were overweight in 2016, with 650 million obese, based on the body mass index (BMI). Obesity is associated with a 48% higher risk of COVID‐19 morbidity and mortality than those whose BMI is within the normal range.^1^

For morbidly obese people, bariatric surgery is the best option for achieving and maintaining weight loss. Bariatric surgery has also been proven more beneficial than standard medical therapy alone in reducing or eradicating type 2 diabetes, hypertension, obstructive sleep apnea, and hyperlipidemia.^2^, ^3^ According to a recent cohort study, prior bariatric surgery is associated with lower in‐hospital mortality rates and mechanical ventilation.^4^ However, bariatric surgery has complications, such as secondary hyperoxaluria, the most prevalent metabolic anomaly discovered in these individuals, with prevalence rates ranging from 29% to 67%.^5^, ^6^, ^7^

**FIGURE 1:**
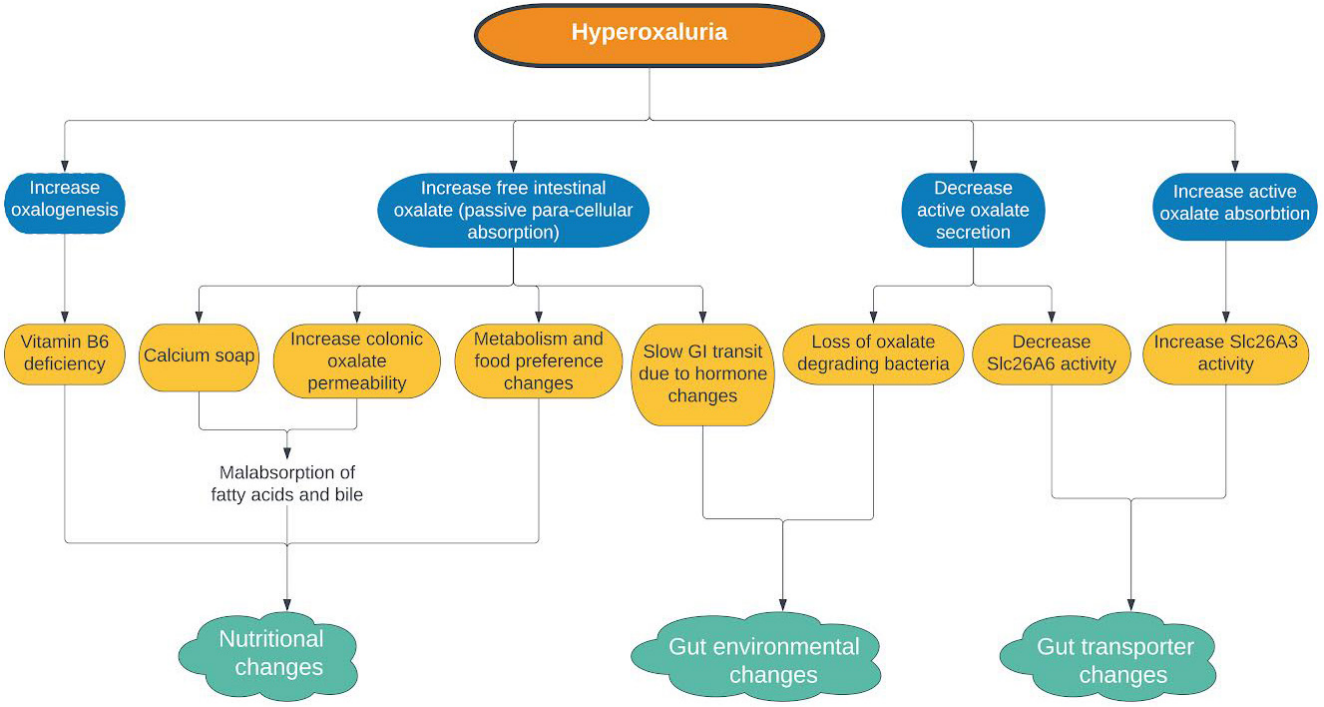
Hyperoxaluria occurred when the amount of free intestinal oxalate, oxalogenesis, and active oxalate absorbed increases and the amount of active oxalate excreted decreases, as seen in this graph.

Oxalate is an organic acid in many plant‐based foods (leafy green vegetables) and plant‐based products (chocolate, peanut butter). It is a result of human liver metabolism produced from various precursors. Around 50–60% of urine oxalate is produced from daily metabolism; the rest is acquired from dietary oxalate, and the kidney is the primary excretory organ. Hyperoxaluria is caused by the presence of soluble oxalate in the intestinal lumen and increased permeability of the colonic mucosa. Oxalate excretion and kidney stone formation increase as oxalate production or intestinal digestion increases.^8^, ^9^ (Figure 1).

Enteric hyperoxaluria (EH) is a type of digestive illness characterized by fat malabsorption (steatorrhea) due to a diminution in the functional surface area of the small intestine. However, intestinal malabsorption, specifically lipids and bile salts, increases oxalate absorption. EH was initially observed in individuals after intestinal resection for inflammatory bowel disease and mesenteric ischemia. Later, reports of hyperoxaluria, urolithiasis, and even kidney failure from oxalate nephropathy demonstrated that EH was also a consequence of modern bariatric surgery. As a result, bariatric surgery is the most common cause of EH.^10^ Also, due to the incidence of COVID‐19 illness and the administration of high‐dose vitamin C supplements, it is essential to know that COVID‐19 and high‐dose vitamin C supplements can cause oxalate nephropathy by endogenous ascorbic acid (AA) to oxalate conversion from vitamin C.^11^

We present a unique occurrence of oxalate nephropathy induced by excessive vitamin C treatment for COVID‐19 prophylaxis in a patient who underwent Roux‐en‐Y gastric bypass (RYGB) 2 years ago.

## 2 CASE PRESENTATION

A 56‐year‐old male was admitted to our nephrology clinic with severe diarrhea and rising creatinine during a routine checkup. His home medications included metformin, metoprolol, and a vitamin C supplement (1 gr/day) for COVID‐19 prevention for almost a year, and there was no family history of kidney disease. Table 1 shows patient demographics.

**TABLE 1:** Patient demographics.

Characteristic	
Sex	Male
Age (year)	58
Renal function (GFR)	38
Diabetes mellitus	Yes
Hypertension	Yes
Chronic obstructive pulmonary disease	No
Smoker	No
Symptomatic kidney stone	No
Alcohol consumption	No

Abbreviation: GFR, Glomerular filtration rate.

He was hemodynamically stable on presentation (blood pressure: 125/80 mmHg, temperature: 37°C, pulse rate: 85 beats/min), and his physical examination, cardiopulmonary, abdominal, and neurologic exams were unremarkable. Initial laboratory studies reveal the serum creatinine level of 1.94 mg/dL, the urea level of 55 mg/dL, and the sodium and potassium levels were normal. One week later, the patient's renal function was mildly worsened (serum creatinine 2.1 mg/dL, urea 65 mg/dL). No cast or crystal was found in the urine tests. Table 2 summarizes preliminary laboratory results and 24‐hour urine test results 3 days after his initial visit.

**TABLE 2:** Initial laboratory data.

Test	Result	Reference range
K (mEq/L)	3.6	3.5–5.5
Na (mEq/L)	138	135–145
Hgb (g/dL)	12.7	11–15
WBC (10^3^/uL)	7400	4000–11,000
Platelets (10^5^/uL)	191	150–450
HCT (%)	36.4	36–50
Creatinine (mg/dL)	1.94	0.6–1.2
Alb (g/dL)	4.3	3.5–5.2
Hb A1C (%)	6.2	3.8–6
FBS (mg/dL)	105	70–100
Uric acid (mg/dL)	55	17–43
Phosphorus (mg/dL)	3.4	2.6–4.5
Calcium (mg/dL)	9.8	8.6–10.3
Urine protein 24 h	65	25–150 mg/24 h
Urine creatinine 24 h	1118	700–2500 mg/24 h
Urine oxalate 24 h	432	100–550 nmol/24 h
Urine citrate 24 h	560	Males: 120–930 mg/24 h Females: 250–1170 mg/24 h
Urine calcium 24 h	116	20–300 mg/24 h
Urine volume 24 h (mL/day)	1300	Adult: 600–1800
Protein/creatinine ratio	0.06	Over 0/3 significant proteinuria
HBS‐Ag (ECL)	0/187	Positive: >1 Negative: <1
HIV Ab + HIV‐1P24 Ag (ECL)	Non‐reactive	
HBS‐Ab (ELISA, mIU/mL)	239.3	Negative: <10 Positive: ≥10
HCV Ab (ECL)	Non‐reactive	
Uric acid (mg/dL) (1 week later)	65	17–43
Creatinine (mg/dL) (1 week later)	2.1	0.6–1.2
ALT	25	5–41 U/L
AST	21	5–38 U/L
ALP	312	100–320 U/L

Abbreviations: ALP, Alkaline Phosphatase; ALT, Alanine transaminase; AST, Aspartate aminotransferase; ECL, ECLusys assay; FBS, fasting blood sugar; HBS‐Ag, Hepatitis B surface antigen; HCT, Hematocrit;HIV, human immunodeficiency virus; HCV, Hepatitis C virus.

Our patient has a history of BPH from 8 years ago, triennial hypertension, diabetes mellitus from 7 years ago, gastric bypass surgery (RYGB with 120 cm jejunum bypass) 2 years earlier, and excessive peanut consumption in the diet. A normal Doppler study and enlarged prostate were also found during a renal ultrasound.

**FIGURE 2:**
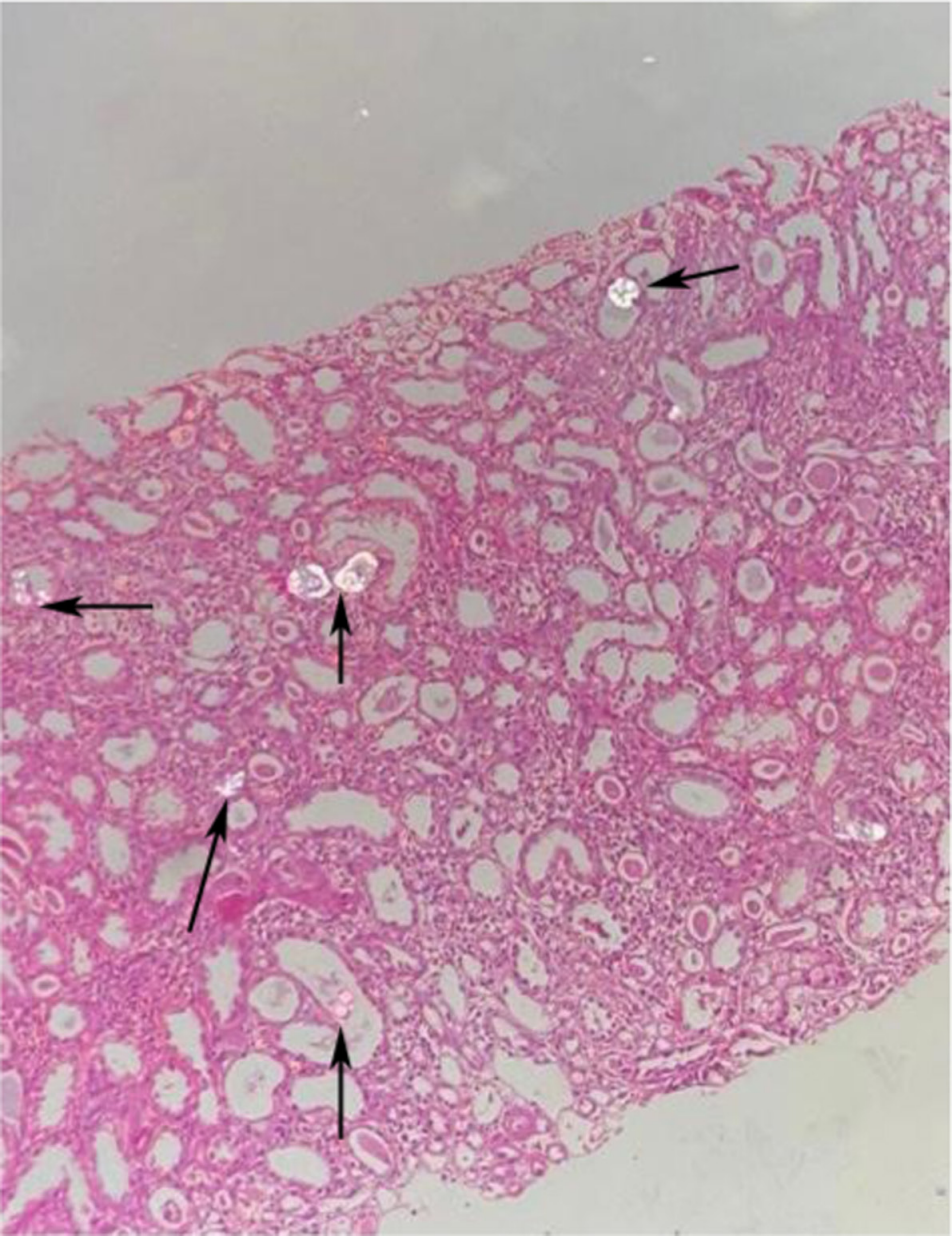
Arrows show the calcium oxalate deposits within renal tubules under polarized light.

**FIGURE 3:**
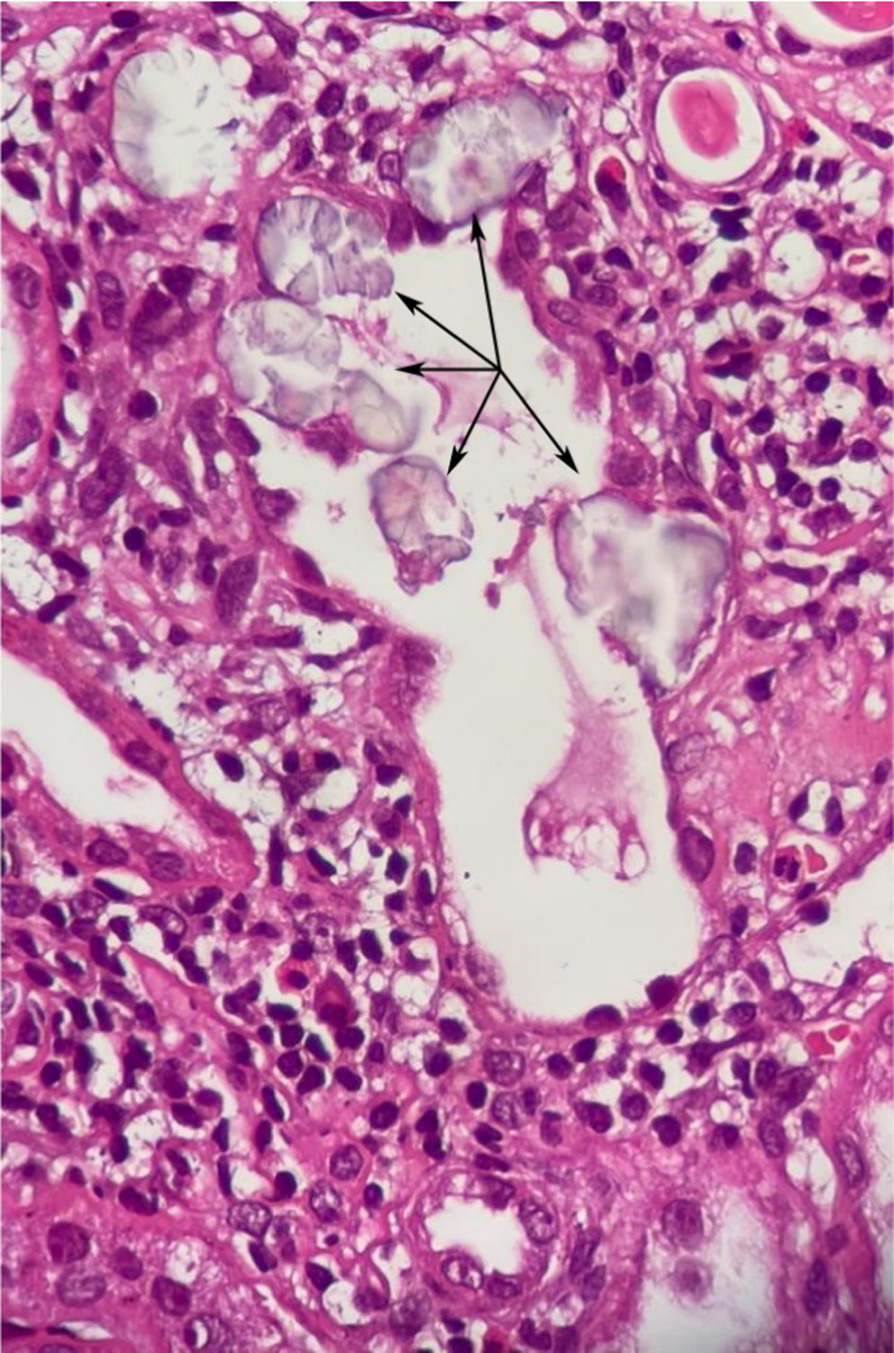
Calcium oxalate deposits within renal tubules are associated with severe tubular injury and mild to moderate interstitial inflammation and edema. H&E stain.

A kidney biopsy was done using three soft tissue cores of 1.7, 1, and 0.6 cm in length for light microscopy and two cores of 1.3 and 1.1 cm in length for immunofluorescence microscopy. Microscopically, there was mild to moderate interstitial fibrosis and tubular atrophy (25%–30%), as well as significant intratubular calcium oxalate crystal aggregation, indicating secondary oxalate nephropathy. Moderate arteriosclerosis and arteriolar hyalinosis are also signs of hypertension‐related vascular alterations (Figures 2 and 3).

Immunofluorescence microscopy was performed to stain frozen sections of 12 glomeruli with IgG, IgA, IgM, C1q, C4, C3, fibrinogen, lambda, and kappa antibodies that revealed a negative immunological reaction.

## 3 FOLLOW‐UP

**FIGURE 4:**
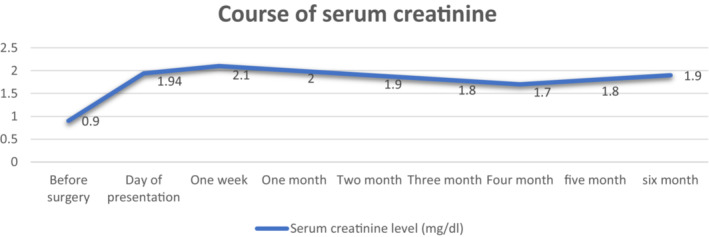
Course of serum creatinine during 6‐month follow‐up.

At the time of Roux‐en‐Y gastric bypass, our patient's serum Cr level was 0.9 mg/dL. The decision to do a laparoscopic reversal of RYGB was made because of the risk of rapid progression to renal failure; however, the patient did not agree. As a result, the patient was recommended to drink more water and limit his peanuts and vitamin C intake. In addition, creatinine and urea clearance tests were carried out monthly to monitor renal function. His serum creatinine level remained stable at 1.7–2 mg/dL. Despite prolonged supportive care, there was no meaningful improvement in renal function after 6 months (Figure 4).

## 4 DISCUSSION AND CONCLUSIONS

We present an interesting case of AKI due to oxalate nephropathy triggered by high consumption of peanuts and daily vitamin C intake (1 g) in a patient with RYGB and several morbid conditions (diabetes, hypertension, BPH). Secondary oxalate nephropathy in our patient could be explained by several underlying factors.

First, enteric alterations caused by RYGB: (1) Following bariatric surgery, free fatty acids bond to intestinal calcium, increasing the amount of unbound oxalate that the gut can absorb. Increased awareness of oxalate nephropathy secondary to RYGB is warranted now more than ever due to the rising tide of bariatric surgery worldwide. In a meta‐analysis and systematic review, a higher incidence of renal stone formation and elevated urine oxalate and calcium oxalate supersaturation has been linked to Roux‐en‐Y gastric bypass surgery.^12^ Duffey et al.^6^ found that after RYGB, there was a statistically significant rise in urine oxalate and that de novo hyperoxaluria occurred in 52% of patients. Another study by Nasr et al.^13^ showed oxalate nephropathy is a side effect of RYGB that does not get enough attention. It should be considered as a possible cause of sudden loss of kidney function after RYGB. As the same result, Sinha et al.^14^ stated that hyperoxaluria is a probable consequence after 12 months following surgery. (2) Free fatty acids and bile salts may directly increase the intestinal permeability to oxalate.^13^

Second, eating oxalate‐rich meals or foods high in oxalate precursors such as vitamin C and peanuts can lead to ingestion‐related hyperoxaluria.^15^ According to Chai et al.^16^ roasted peanuts have a mean oxalate content of 140 mg/100 g. A few studies have described peanut‐induced oxalate nephropathy. Park et al.^17^ state a case with oxalate nephropathy due to consumption of peanuts (>130 g/day) that visited the emergency room for persistent vomiting and diarrhea. Also, the same issue presented by Sasaki et al.^18^ due to excessive intake of peanuts with fever and renal dysfunction with mild diabetes mellitus. Froeder et al.^19^ state that oxalate in the diet can worsen intestinal hyperoxaluria in patients who had bariatric surgery. Vitamin C has no known hazardous dosage, but 1000 mg/d can increase oxalate excretion.^20^ Some Studies found excessive daily ingestion of high‐dose vitamin C intake linked to renal failure. Auer et al.^21^ illustrate a 25‐year‐old person who consumed 8 g of AA over 8 days. After that, he had increased oxalate excretion and crystalluria. In addition, Fontana et al.^11^ report two cases of iatrogenic AKI caused by vitamin C supplementation in the context of COVID‐19 and without a clear causative link to SARS‐CoV‐2 infection. Besides consuming a lot of vitamin C for the COVID‐19 prevention strategy, our patient used a lot of peanuts in his diet.

Third, BPH may have contributed to increased crystal deposition in the renal tubules due to persistent urine retention.^22^ Also, Lin et al.^23^ presented a 69‐year‐old male patient with a history of BPH, small bowel resection, and taking 2 g of vitamin C every day for almost 2 years who presented with acute kidney injury due to oxalate nephropathy. Also, our patient had a preexisting condition of BPH, which may have influenced the accumulation of oxalate crystals.

Fourth, individuals with a history of diabetes have elevated plasma levels of glyoxylate and glyoxal, which can result in hyperoxaluria.^24^

Based on this case report and the research discussed previously, RYGB patients require long‐term monitoring of renal function and dietary modifications. Diagnosing kidney impairment early on is crucial, as it may be possible to reverse the damage through surgery.^25^ Patients who have had RYGB should also be aware of the risks associated with high doses of vitamin C with substances that contain high doses of oxalate, according to the current COVID‐19 pandemic. Additionally, clinicians should consider oxalate nephropathy in the differential diagnosis of AKI in patients with RYGB. Prescription of high doses of vitamin C should be avoided in these patients and dietary counseling concerning de danger of ingestion of high oxalate products.

## AUTHOR CONTRIBUTIONS

**Fatemeh kafi:** Writing – original draft; writing – review and editing. **Mojgan Mortazavi:** Resources; supervision. **Alireza Pouramini:** Data curation; visualization; writing – original draft; writing – review and editing. **Shahaboddin Dolatkhah:** Writing – review and editing. **Behrouz Kaleidari:** Conceptualization; supervision. **Diana Taheri:** Conceptualization; investigation; supervision.

## CONFLICT OF INTEREST STATEMENT

There are no conflicts of interest reported by the authors. The authors are solely responsible for the article's content and writing.

## CONSENT

Written informed consent was obtained from the patient to publish this report in accordance with the journal's patient consent policy.

## Data Availability

Data sharing not applicable to this article as no datasets were generated or analysed during the current study
